# Mapping the spatial and temporal frequency of systemic lupus erythematosus in Brazil

**DOI:** 10.1590/1980-549720250030

**Published:** 2025-06-02

**Authors:** Andreza Martyres, Alice Ramos-Silva, Fabiana Rabe Carvalho, Rodrigo Cutrim Gaudio, Katia Lino Baptista, Elisangela Costa Lima, Thalia Medeiros, Andrea Alice Silva

**Affiliations:** I Universidade Federal Fluminense, Faculty of Medicine, Multiuser Laboratory for Research in Nephrology and Medical Science – Niterói (RJ), Brazil.; II Universidade Federal Fluminense, School of Medicine, Department of Pathology – Niterói (RJ), Brazil.; III Universidade Federal Fluminense, Hospital Universitário Antônio Pedro, Rheumatology unit – Niterói (RJ), Brazil.; IV Universidade Federal do Rio de Janeiro, Faculty of Pharmacy – Rio de Janeiro (RJ), Brazil.

**Keywords:** Lupus erythematosus, systemic, Prevalence, Brazil, Spatio-temporal analysis, Public health, Lúpus eritematoso sistêmico, Prevalência, Brasil, Análise espaço-temporal, Saúde pública

## Abstract

**Objective:**

The aim of this study was to analyze the spatial and temporal distribution of systemic lupus erythematosus (SLE) cases in Brazil from 2008 to 2022.

**Methods:**

We conducted an ecological study based on data from patients treated in the Unified Health System. SLE cases were identified using International Classification of Diseases-10 codes and analyzed by geographic region, age, and color/race. Spatial distribution was assessed to identify high and low prevalence, while temporal trends were evaluated through annual percentage change (APC).

**Results:**

In 2022, the national prevalence was 52.3/100,000 inhabitants, with marked geographical disparities. Southeast (68.14/100,000) and South (66.37/100,000) regions showed the highest reporting rates. Spatial analysis identified significant clustering, particularly in São Paulo and Paraná, accounting for 95.4% of the high-prevalence municipalities. Temporal analysis of the adult population revealed a consistent increase in SLE prevalence from 2008 to 2022 (APC=15.5%, p<0.001), which was most pronounced in the Northeast and South, while a slower increase was observed in the North. A correlation was observed between the number of rheumatologists and the number of cases/100,000 inhabitants (R=0.567, p=0.002).

**Conclusion:**

This study reveals significant geographic disparities and a rising trend in SLE prevalence across Brazil. The clustering of cases in specific municipalities and the correlation between rheumatologist availability and prevalence underscore the need for targeted healthcare resources. These findings highlight the importance of investigating how healthcare access impacts regional disparities in SLE prevalence and advancing equitable care nationwide.

## RESUMO

**Objetivo:**

Analisar a distribuição espacial e temporal dos casos de lúpus eritematoso sistêmico (LES) no Brasil de 2008 a 2022.

**Métodos:**

Estudo ecológico baseado em dados dos usuários do Sistema Único de Saúde. Os casos de LES foram identificados pela 10ª edição da Classificação Internacional de Doenças e Problemas Relacionados à Saúde e analisados por região, idade e etnia. A análise espacial avaliou *clusters* de prevalência, enquanto a variação percentual anual foi estimada por região e grupo etário.

**Resultados:**

Em 2022, a prevalência nacional foi de 52,3/100 mil habitantes, com disparidades regionais. As regiões Sudeste (68,14/100 mil) e Sul (66,37/100 mil) apresentaram maiores taxas. *Clusters* significativos foram encontrados em São Paulo e Paraná, abrangendo 95,4% dos municípios com alta prevalência. A análise temporal dos dados da população adulta revelou aumento consistente na prevalência do LES de 2008 a 2022 (VPA=15,5%, p<0,001), especialmente no nordeste e sul. O crescimento foi mais lento no norte. Houve correlação moderada entre o número de reumatologistas e casos por 100 mil habitantes (R=0,567, p=0,002).

**Conclusão:**

O estudo destaca disparidades regionais e a tendência crescente na prevalência de LES no Brasil. *Clusters* em municípios específicos e a relação com a disponibilidade de reumatologistas evidenciam a necessidade de alocar recursos de forma direcionada. Esses achados reforçam a importância de se investigar como o acesso aos cuidados de saúde influencia as disparidades e de promover equidade no atendimento em todo o país.

## INTRODUCTION

Systemic lupus erythematosus (SLE) is a chronic autoimmune disease characterized by a relapsing-remitting course, with periods of clinical remission and exacerbation^1^. The etiology of SLE remains incompletely understood; however, it is recognized as a multifactorial condition with a heterogeneous distribution influenced by a complex interplay of genetic predisposition, racial/ethnic background, environmental exposures, hormonal factors, infectious agents, and socioeconomic determinants^2-5^. Recent studies have implicated those environmental factors, such as air pollutants and ultraviolet B radiation, are also involved in the pathogenesis of SLE through mechanisms such as DNA hypomethylation in CD4+ T cells, which may contribute to disease activity and increased hospitalization risk in juvenile populations^6-8^.

SLE global incidence and prevalence vary across different geographic regions, largely driven by disparities in socioeconomic status, healthcare access, and diagnostic practices^9^. Historically, it was assumed that there was lower SLE prevalence in African populations compared to Europeans^10^. However, recent epidemiological data indicate that African/Asian descent present a two- to three-fold higher SLE prevalence when compared to Caucasians, with blacks and Hispanics experiencing greater morbidity and mortality^11^. These disparities underscore the important role of social determinants of health in the burden of chronic diseases, particularly in low- and middle-income countries^12^.

Brazil, as the fifth largest country in the world, presents extensive geographical and ethnic diversity^13^. Despite this, comprehensive national data on the epidemiology of SLE are scarce, with few studies limited to localized areas and small populations. This lack of data jeopardizes the development of effective regional health strategies and public policies tailored to address the specific needs of the Brazilian population. To fill this gap, the present study aims to provide a detailed analysis of the spatial and temporal distribution of SLE cases in Brazil from 2008 to 2022. By elucidating the epidemiological landscape across the country’s federative units, this study seeks to support the development of targeted interventions and improve healthcare planning and resource allocation for SLE management in Brazil.

## METHODS

### Study design

We carried out an ecological study using data from patients treated within the Unified Health System (SUS). The study aimed to assess the prevalence of systemic lupus erythematosus (SLE) based on administrative and health records over a 15-year period. Brazil spans approximately 8.5 million square kilometers and has a population of around 203 million. The country is administratively divided into five macro-regions (North, Northeast, Central-West, Southeast, and South), 27 federative units, and 5,570 municipalities.

### Variables and data sources

Data was obtained from two primary systems: the Outpatient Information System (SIA) and the Hospital Information System (SIH), which record information on all patients using the SUS. While data from private hospital admissions and patients with SLE treated in private practices are excluded, these systems offer essential nationwide insights. They support evidence-based government actions, track compliance with international health indicators, and enable the publication of technical, epidemiological, and scientific studies^14^. This study included patients diagnosed with SLE, identified using the following International Classification of Diseases (ICD-10) codes: M320, M321, M328, and M329 (indicating lupus with musculoskeletal or connective tissue involvement) and L93, L930, L931, and L932 (indicating various forms of cutaneous lupus erythematosus). The following variables were analyzed to assess SLE prevalence and distribution:

- Patients treated in SUS: The number of SLE patients treated in the SUS network was expressed as standardized rates per 100,000 inhabitants. Population data for each location were obtained from the Brazilian Institute of Applied Statistics. Outpatient data were sourced from SIA/SUS and inpatient data from SIH-SUS. In this study, “prevalence” refers to the total number of outpatients in the SUS. Inpatients and outpatients were age-standardized using the direct method, with the total population of each age group as the reference (e.g., SLE cases in children under 10 years divided by the total population under 10 years). This method ensures comparability across municipalities, federative units, and regions by accounting for differences in age distribution and minimizing potential biases due to population structure variations;
- Age stratification: Subgroup analyses were initially conducted for patients under 19 years of age to assess age-specific prevalence and distribution. This cutoff was chosen to align with international classifications that distinguish pediatric (<19 years) from adult (≥19 years) populations. Additional analyses were performed using broader age categories: children (<10 years), adolescents (10–19 years), adults (20–59 years), and the elderly (≥60 years). These groupings provided a more comprehensive understanding of prevalence and distribution patterns across age groups;
- Systemic disease subgroup: Further analyses were performed for patients with systemic manifestations of lupus (ICD-10 M320, M321, M328, and M329);
- Spatial data: Geographic data were integrated using the cartographic database from the Brazilian Institute of Geography and Statistics^13^ to evaluate spatial patterns in SLE prevalence;
- Population data: Population estimates from Instituto Brasileiro de Geografia e Estatística (IBGE) were used to calculate prevalence rates and to provide context for SLE distribution across different regions;
- Color/race data: Color/race information, as recorded in SIA, was collected based on self-declaration by the patient or their legal guardian;
- Rheumatology services: were assessed by the number of rheumatology specialist physicians, standardized per 100,000 inhabitants in each geographic region. The data on medical specialties were obtained from the National Registry of Health Establishments (*Cadastro Nacional de Estabelecimentos de Saúde*);
- The mortality rate: was calculated as the number of deaths divided by the total number of hospital admissions for SLE. These data were extracted from the SIH-SUS database.

### Data analysis

Variables are reported as absolute counts and relative frequencies. Case rates and physician numbers were standardized per 100,000 population. The Shapiro-Wilk test was applied to guide test selection. Pearson’s correlation test assessed the relationship between the number of rheumatologists and SLE cases. In-hospital mortality rates for SLE patients were analyzed using the χ^2^ test. Kruskal–Wallis test compared outpatient treatment rates for SLE patients across age groups: children, adolescents, adults, and the elderly.

Spatial distribution maps were generated using R software (version 4.3.2) and the cartographic data provided by IBGE. Spatial dependence, which refers to the extent to which SLE prevalence in one area influences neighboring areas, was assessed using spatial autocorrelation metrics.

A first-order contiguity matrix was constructed, where municipalities sharing borders were considered neighbors (value=1) and those without shared borders were considered non-neighbors (value=0). Spatial autocorrelation was initially evaluated using Global Moran’s I statistic. Local spatial clusters were identified using Local Indicators of Spatial Association (LISA), enabling the detection of regions with significant spatial patterns, such as “high-high” (clusters with high prevalence) and “low-low” (clusters with low prevalence) areas. Statistical significance was set at p≤0.05 for all analyses.

Temporal trends in SLE prevalence were assessed using joinpoint regression analysis, which models data using segmented regression with variance estimation via Poisson regression. The significance of trend changes was evaluated using the Monte Carlo permutation method, assuming constant variance (homoscedasticity) and first-order autocorrelation. For each trend segment, the annual percentage change (APC) was calculated and tested against the null hypothesis of no change. Joinpoints represented statistically significant shifts in the direction of the trend, with each segment characterized by its respective APC.

## RESULTS

**Figure 1:**
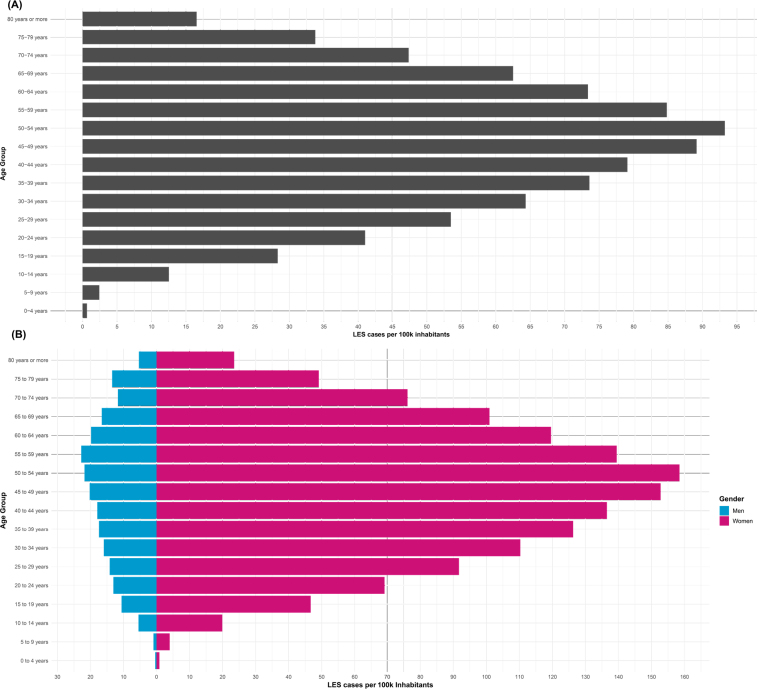
Prevalence rate of systemic lupus erythematosus cases per 100,000 inhabitants in Brazil by age in 2022: (A) Total cases in the Brazilian population by age. (B) Total cases by age and sex.

In 2022, a total of 106,156 patients were registered in the outpatient system for an SLE diagnosis in Brazil, corresponding to a prevalence of approximately 52.3 cases per 100,000 inhabitants, based on a total population of 203,062,512. The majority of patients were female (N=92,843; 87.5%), with a median age of 44 years (interquartile range: 33–55 years). Regarding racial identity, 36,424 (34.3%) individuals identified as white, 32,684 (30.8%) as mixed races, and 37,069 (34.9%) did not report their racial or ethnic background. Age-specific analysis revealed a lower prevalence of SLE among children under 9 years old, with an estimation of three cases per 100,000 inhabitants. We observed a higher prevalence of SLE, peaking at 90–95 cases per 100,000 inhabitants among individuals aged 45–54 years (Figure 1). Adult and elderly patients showed significantly higher rates of SLE compared to other age groups (p<0.0001).

**Figure 2:**
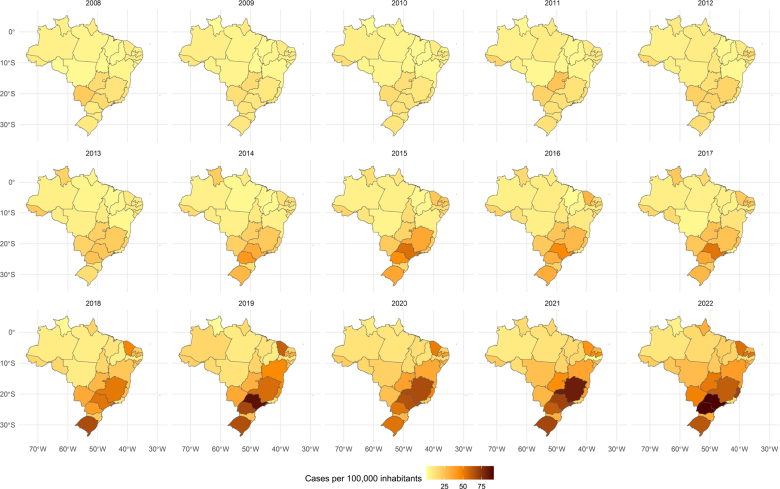
Geographic distribution of systemic lupus erythematosus (SLE) prevalence in Brazil by year.

In 2022, the prevalence of SLE and lupus erythematosus (LE) significantly varied across the Brazilian macro-regions. The highest prevalence rates were observed in the Southeast (68.14 cases per 100,000 inhabitants) and South (66.37 cases per 100,000 inhabitants), followed by the Midwest (43.92 cases per 100,000 inhabitants) and Northeast (33.33 cases per 100,000 inhabitants). In contrast, the North region had the lowest prevalence, with 15.38 cases per 100,000 inhabitants (Figure 2). The Moran’s I statistic, calculated to assess spatial autocorrelation of SLE prevalence per 10,000 inhabitants, indicated a significant positive spatial autocorrelation (Moran’s I=0.3177, p<0.0001), suggesting a non-random geographical distribution of cases.

Further analysis using LISA identified distinct clusters of SLE. Six states (Maranhão, Minas Gerais, Rio de Janeiro, São Paulo, Paraná, and Rio Grande do Sul) and 240 municipalities exhibited “high-high” clusters, indicating areas with high prevalence positively influencing neighboring areas. Conversely, five states (Pernambuco, São Paulo, Paraná, Santa Catarina, and Rio Grande do Sul) and 39 municipalities showed “low-low” clusters, representing regions with low prevalence negatively influencing neighboring areas. Notably, two states—São Paulo and Paraná—accounted for 95.4% (n=229) of the municipalities classified as “high-high” clusters.

When the analysis was restricted to SLE cases alone (ICD-10 M320, M321, M328, and M329), the regional distribution pattern remained similar. The Southeast and South regions showed the highest prevalence rates (60.64 and 60.46 cases per 100,000 inhabitants, respectively), followed by the Midwest (39.98 cases per 100,000 inhabitants), Northeast (30.32 cases per 100,000 inhabitants), and North (14.64 cases per 100,000 inhabitants).

**Figure 3:**
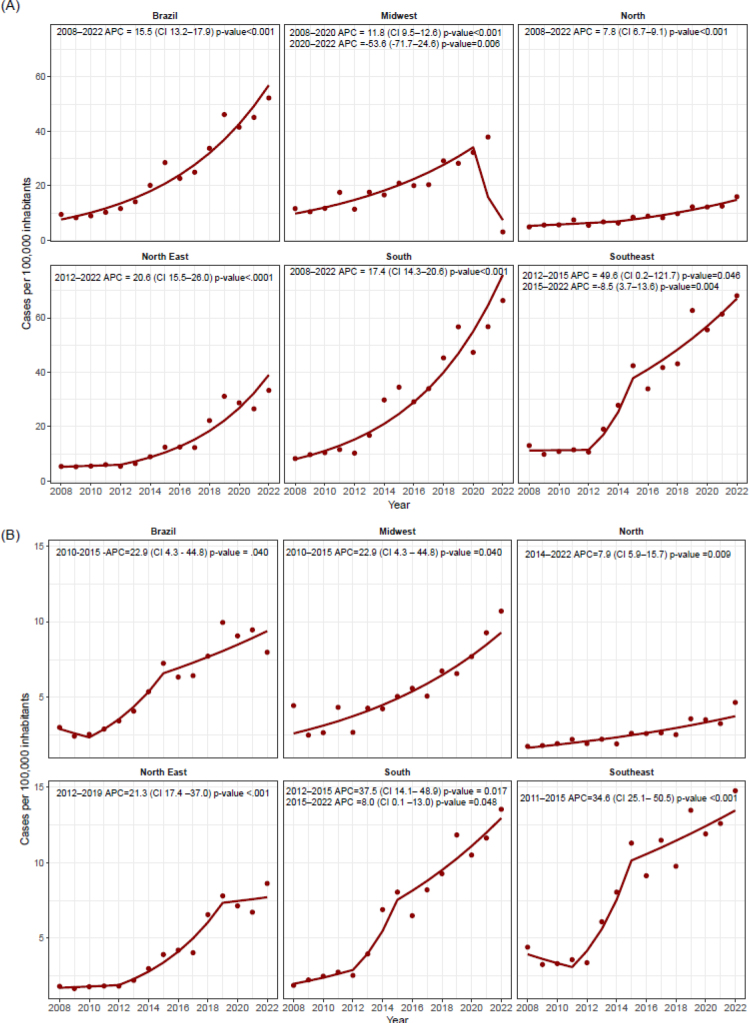
The joinpoint regression indicating the SLE cases rate from 2008 to 2022 per Brazilian region. (A) Data concerning adults, (B) data concerning young adults<19 years of age. Annual percent change (APC).

The temporal analysis of SLE prevalence in adults from 2008 to 2022 revealed a significant upward trend in Brazil, with an APC of 15.5% (p<0.0001). The Northeast and South regions displayed similar trends, with APCs of 20.6 and 17.4%, respectively (both p<0.0001). In contrast, the North region exhibited a more gradual increase over the same period, with an APC of 7.8% (p<0.0001). The Midwest region experienced a notable increase from 2008 to 2021 (APC=11.8%; p<0.0001), which was disrupted in 2020 with a sharp decline (APC=-53.6%; p=0.0060). In the Southeast, a marked rise was observed between 2012 and 2015 (APC=49.6%; p=0.46), followed by a modest decrease in SLE cases between 2015 and 2022 (APC=-8.5%; p=0.0040) (Figure 3A).

Among individuals under 19 years of age, an increasing trend in SLE prevalence was observed between 2010 and 2015 (APC=22.9%; p=0.0400), sustained from 2015 onwards. The temporal patterns in this younger age group varied across the macro-regions. In the Midwest, there was an initial exponential increase (APC=-22.9%; p=0.0400), which plateaued after 2019. Similarly, the Northeast showed an exponential rise in cases (APC=21.3%; p<0.0001) (Figure 3B).

Finally, we analyzed the distribution of rheumatology services across Brazilian states. The number of rheumatologists per 100,000 inhabitants in the federative units ranges from 0.24 to 1.98. The Federal District (DF) has the highest density, with 1.99 rheumatologists per 100,000 inhabitants, followed by Rio de Janeiro (RJ) with 1.31 and Espírito Santo (ES) with 1.15. Conversely, the states with the lowest density of rheumatologists include Acre (AC) with 0.24, Amapá (AP) with 0.27, and Rondônia (RO) with 0.47.

**Figure 4:**
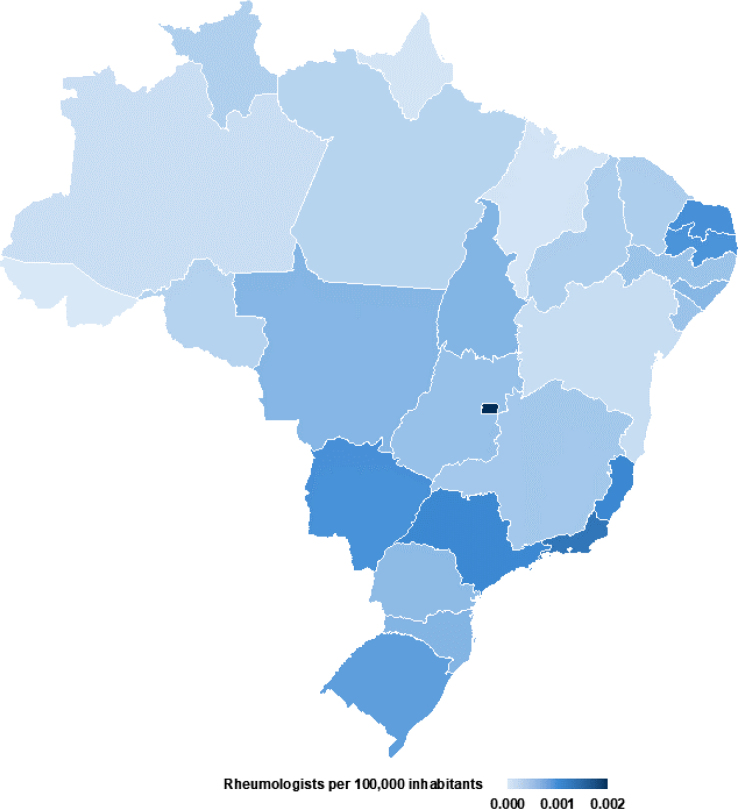
Geographic distribution of rheumatologists per 100,000 inhabitants in Brazil.

The distribution of rheumatologists in Brazil is uneven, with higher concentrations in the South and Southeast and lower access in the North and Northeast, reflecting healthcare inequities for rheumatic disease patients (Figure 4). The Pearson correlation coefficient between rheumatologist density and patient numbers per 100,000 inhabitants was 0.567 (p=0.0020), indicating a moderate positive correlation. States with more rheumatologists tend to have more patients, with a 95% confidence interval of 0.238–0.779. Regional disparities in mortality rates are significant (p=0.0080), with the Midwest having the highest rate (6.72%), followed by the North (4.47%). Brazil’s overall mortality rate is 3.87% (Table 1; Figure 4).

**Table 1:** Prevalence of systemic lupus erythematosus (SLE) cases and number of rheumatologists per region of Brazil (per 100,000 inhabitants) in 2022.

	North	Northeast	Southeast	South	Midwest
**Outpatient SLE cases**	15.960	33.334	68.142	66.367	45.949
Children (<10 years)	0.969	2.101	2.749	2.248	1.799
Adolescents (10–19 years)	9.418	17.243	31.273	28.713	23.301
Adults (20–59 years	26.383	53.459	105.192	99.669	70.571
Elderly (>60 years)	10.668	25.332	58.339	66.914	44.629
**Number of rheumatologists**	2.525	3.870	8.606	6.331	6.799
**Inpatient SLE cases**	0.003	2.063	3.829	3.784	3.290
Children (<10 years)	0.899	1.849	2.34	1.977	2.018
Adolescents (10–19 years)	0.793	3.504	2.739	2.350	3.551
Adults (20–59 years	2.191	3.906	3.829	2.896	2.765
Elderly (>60 years)	6.633	7.517	6.861	7.645	8.135
**Hospital mortality rate (%)**	4.47	4.06	3.46	3.04	6.72

## DISCUSSION

A national prevalence of 52.3 cases per 100,000 inhabitants was observed, with marked heterogeneity among the five macro-regions. The variation in SLE prevalence in Brazil aligns with findings from other countries, such as Argentina (58.6 cases per 100,000 inhabitants), Turkey (51.7 cases per 100,000 inhabitants), and several African nations (60 cases per 100,000 inhabitants)^15-18^. The age-stratified prevalence was also consistent with reports in the literature, showing lower rates in children compared to adults^19^. The density of rheumatologists in Brazil highlights disparities that affect SLE detection and treatment. A moderate positive correlation between rheumatologist density and SLE prevalence shows that areas with more specialists report more cases. This suggests that increased specialist availability may improve diagnosis, contributing to regional variations in SLE prevalence.

In Brazil, the highest SLE prevalence rates were found in the Southeast (68.14 cases per 100,000) and South (66.37 cases per 100,000), reflecting geographical disparities influenced by socio-economic factors. These regions, with higher gross domestic product and better healthcare access, likely see higher detection rates. São Paulo and Paraná, key economic centers, accounted for 95.4% of municipalities classified as “high-high” clusters, highlighting the role of economic and healthcare resources in SLE prevalence reporting^20,21^.

Environmental factors like ultraviolet (UV) radiation, air pollution, and genetics are triggers for SLE. Prolonged UV exposure creates a pro-inflammatory environment and triggers apoptosis in SLE patients^22^. Caucasians exposed to the sun for over 24 months have three times the risk of developing SLE. In Brazil, high UV radiation levels are common, especially in the summer^15,23^.

From 2008 to 2022, SLE prevalence steadily increased in Brazil, unlike in Europe, where studies in Denmark^24^ and Norway^25^ showed stable incidence over 8 and 10 years, respectively. A long-term study in Spain noted an increase before stabilization^26^, and a 43-year study in the U.S. found a modest 2% rise, with five cases per 100,000, much lower than Brazil^27^ (52.3 cases per 100,000). This suggests that improved diagnostics and awareness may partly explain the rise, but genetic, environmental, and socio-economic factors also play a role. The trend highlights the need for targeted public health interventions and further research.

In 2020, the World Health Organization declared the COVID-19 pandemic, which may have contributed to either an increase or a decrease in the estimated prevalence of SLE. A Greek study found an increase in autoimmune rheumatic diseases from 2020 to 2023 compared to previous years^28^. Factors such as stress and viral infections may have triggered the development of SLE^29^, potentially contributing to the high rate of cases during this period in Brazil’s public health system.

The COVID-19 pandemic impacted Brazil’s healthcare system differently across regions, with the North and Northeast experiencing greater challenges due to socioeconomic disparities and health inequalities. These regions were identified as major risk clusters for COVID-19 mortality, likely affecting the diagnosis of chronic diseases like SLE, which require specialized care and advanced health technologies^30^. Higher SLE rates were observed in the South and Southeast, while lower rates in the North and Northeast may be attributed to limited federal funding and a shortage of rheumatologists. The pandemic also led to an uneven distribution of financial resources, with states like Minas Gerais and São Paulo receiving more support. In contrast, Northern states had fewer resources, forcing them to prioritize COVID-19 management^31^. This reallocation of resources, coupled with the increased mortality risk among SLE patients due to COVID-19, may explain the significant decline in reported SLE cases in the Central-West region between 2020 and 2022. This observation highlights the complex interplay between the pandemic, healthcare access, and SLE incidence, suggesting that while COVID-19 may biologically increase the risk of SLE, it may also lead to an underestimation of its prevalence due to healthcare disruptions.

Our study has several limitations. First, we relied on administrative data, which limited access to clinical and epidemiological variables, hindering the development of a more detailed patient profile^32^. Additionally, we focused only on patients treated within the public healthcare system, which may underestimate the true prevalence of SLE, as it is not a notifiable disease and data on private healthcare patients are unavailable. However, since 71.5% of the Brazilian population uses the public system for chronic disease treatment, our findings still capture a significant portion of SLE cases in Brazil^20,21^. Secondary data are also prone to errors, and the SIH aggregates data by total admissions without distinguishing individual patients^32^, potentially impacting the accuracy of our results. Previous studies have shown that data quality varies by region, particularly in low-income areas^33^. The lower SLE rates observed in the North may be explained by this disparity in data quality. SLE treatment is government-funded and impacts the national health budget; the data in this study can inform health policy planning and highlight areas requiring further investigation^34,35^.

Furthermore, the statistical methods employed have inherent limitations. Joinpoint regression provides a robust framework for detecting significant changes in temporal trends by identifying specific points (joinpoints) where the direction or magnitude of a trend shifts. This method is particularly useful for analyzing temporal patterns, such as periods of increase, decrease, or stability, and for calculating the APC within each segment. However, it assumes that the data follow a Poisson distribution and that variance remains constant (homoscedasticity), which may not always align with the characteristics of all datasets. Additionally, while effective in identifying trend changes, this method does not account for external covariates or confounding factors that may influence the observed patterns. Despite these limitations, joinpoint regression remains a widely used tool for describing temporal changes in epidemiological data and supporting public health analyses^36^.

Despite the limitations, our study presents the first comprehensive analysis of the epidemiology of SLE in Brazil, revealing significant geographic and temporal disparities that warrant further investigation. From 2008 to 2022, the prevalence of SLE increased consistently, with the highest rates observed in the Southeast and South, where areas of high prevalence were concentrated. The moderate correlation between the density of rheumatologists and the prevalence of SLE underscores the role of access to healthcare and highlights regional inequalities that may impact patient outcomes.

## Data Availability

The study utilized anonymized, publicly available secondary data and therefore did not require evaluation by an ethics and research committee.
